# The association between cardiovascular health and obstructive sleep apnea symptoms: findings from NHANES

**DOI:** 10.3389/fcvm.2024.1466752

**Published:** 2024-12-20

**Authors:** Qian Guo, Dong Dong, Qiang Zhou, Shuman Huang, Xinjie Qiao, Zihan Dang, Xiaowu Wang, Yulin Zhao

**Affiliations:** ^1^ Department of Rhinology, The First Affiliated Hospital of Zhengzhou University, Zhengzhou, China; ^2^ Department of Burn and Skin Repair, The Third Affiliated Hospital of Wenzhou Medical University (Ruian People’s Hospital), Wenzhou, China; ^3^ Department of Health Studies & Applied Educational Psychology, Columbia University, New York, NY, United States

**Keywords:** obstructive sleep apnea symptoms, cardiovascular health, NHANES, life's essential 8 metrics, restricted cubic splines

## Abstract

**Objective:**

To investigate the association between cardiovascular health (CVH) and obstructive sleep apnea (OSA) within the U.S. population.

**Methods:**

This study enrolled 12,540 participants aged 20 years and older from the 2007–2008 and 2015–2018 cycles of the National Health and Nutrition Examination Surveys (NHANES). Weighted univariate and multivariate logistic regression were utilized to examine the relationship between CVH and OSA symptoms. Life's Essential 8 (LE 8) metrics was employed to evaluate the CVH status of participants. Identification of OSA symptoms was determined based on a sleep questionnaire. They include (1) how often you snore; (2) how often you snort/stop breathing; or (3) how often you feel overly sleepy during day. Individuals who answered that they snore 3 or more per week; snort/stop breathing 3 or more per week and feel overly sleepy during day 16–30 times per month were classified as having OSA symptoms.

**Results:**

Significant inverse associations were observed between LE8 scores and symptoms of OSA after adjusting for covariates. The 95% CI was 0.750 (0.630,0.893) for the moderate CVH group and 0.573 (0.454,0.723) for the high CVH group. Subgroup analyses, stratified by age and gender, highlighted a significant interaction between LE8 scores and OSA symptoms with age (*P* < 0.0001). Participants under 60 years old in the high CVH group exhibited a reduced likelihood OSA symptoms (OR: 0.470; 95% CI: 0.345,0.641). Restricted cubic splines (RCS) in a multivariate regression analysis showed a non-linear relationship between LE8 score and OSA. Our finding demonstrates a substantial decrease in OSA symptom prevalence with increased LE 8 scores.

**Conclusion:**

The results demonstrate a strong inverse correlation between LE8 scores and OSA symptoms. Participants with higher LE8 scores showed a reduced likelihood of experiencing OSA symptoms.

## Introduction

Obstructive sleep apnea (OSA), or obstructive sleep apnea hypopnea syndrome (OSAHS), is a condition in which patients suffer from repeated apnea and hypoventilation during sleep (1). Common clinical symptoms of OSA can often include loud and irregular snoring, choking or suffocating awakenings at night, sleep disturbances, daytime drowsiness, memory loss, and, in severe cases, cognitive decline and behavioral abnormalities (2). Researcher widely recognize OSA as a systemic disease that serves as an independent risk indicator for hypertension. It is closely linked to coronary atherosclerotic heart disease (CHD), heart failure, arrhythmias, and diabetes, and is a major cause of sudden death and road traffic accidents (3–6). Inflammation and oxidative stress are the biological mechanisms underlying the relationship between OSA and its various health complications (7). In the United States, about 26 percent of people aged between 30 and 70 receive an OSA diagnosis, and the prevalence of OSA increased as individual ages increased (8, 9). Moreover, eleven population-based epidemiological studies reported 22% OSA in males and 17% OSA in females (10). As a result, OSA is considered a threat to public health and a serious social problem.

Medical and epidemiological studies often employ cardiovascular health(CVH) to describe the level of cardiovascular disease risk in an individual or population (11, 12). The assessment often considers several risk factors, such as blood pressure, cholesterol levels, diabetes, tobacco use, dietary intake, physical activity, and body mass. As one of the most common respiratory system diseases, OSA, one of the most common respiratory system diseases, may induce hypertension and contribute to CHD, nocturnal angina, myocardial infarction, severe arrhythmias at night, psychiatric abnormalities, respiratory failure, secondary erythrocytosis, and increased blood viscosity (13). The high prevalence of shared risk factors between OSA and CVH implies a potential synergistic interaction between these diseases, potentially promoting either the initiation or progression of OSA; however, the intricate relationship remains unclear. Therefore, there is an urge to elucidate the intricate relationship of CVH with OSA symptoms.

Life's Essential 8 (LE 8) is the American Heart Association's(AHA) suggested algorithm to measure CVH, which serves as the vital evaluation for promoting and sustaining cardiovascular health (14). In 2022, the AHA updated LS7 to Life's Essential 8 to better address the complexities of modern health conditions and the living environment. Numerous studies have extensively studied LS7 metrics and consistently demonstrated an inverse correlation with coronary heart disease(CHD). Recent studies have established a clear inverse relationship between LE8 scores and the risks of cardiovascular disease (CVD) (15).

We performed a cross-sectional analysis utilizing data from the National Health and Nutrition Examination Survey (NHANES) to examine the correlation between CVH and OSA symptoms. Our findings suggested a potential association between LE 8 scores and OSA symptoms, even after adjustment for potential confounding factors.

## Methods

### Data source

NHANES, overseen by the Centers for Disease Control and Prevention (CDC), surveys the health and nutritional status of adults and children in the US with special emphasis on disease prevalence, adverse risk factors to health, and dietary intake (16). Public health research and policy is heavily dependent on NHANES data for understanding the overall population health, epidemiology of disease trends, and taking action (17). All NHANES procedures were conducted per the guidelines of The Ethical Review Committee at the National Centre for Health Statistics, and written informed consent from participants was obtained before initiation. This study adhered to the Strengthening the Reporting of Observational Studies in Epidemiology (STROBE) guidelines for reporting cross-sectional studies (18). Thus, this study is not required for additional institutional review board approval.

### Study design and population

**Figure 1:**
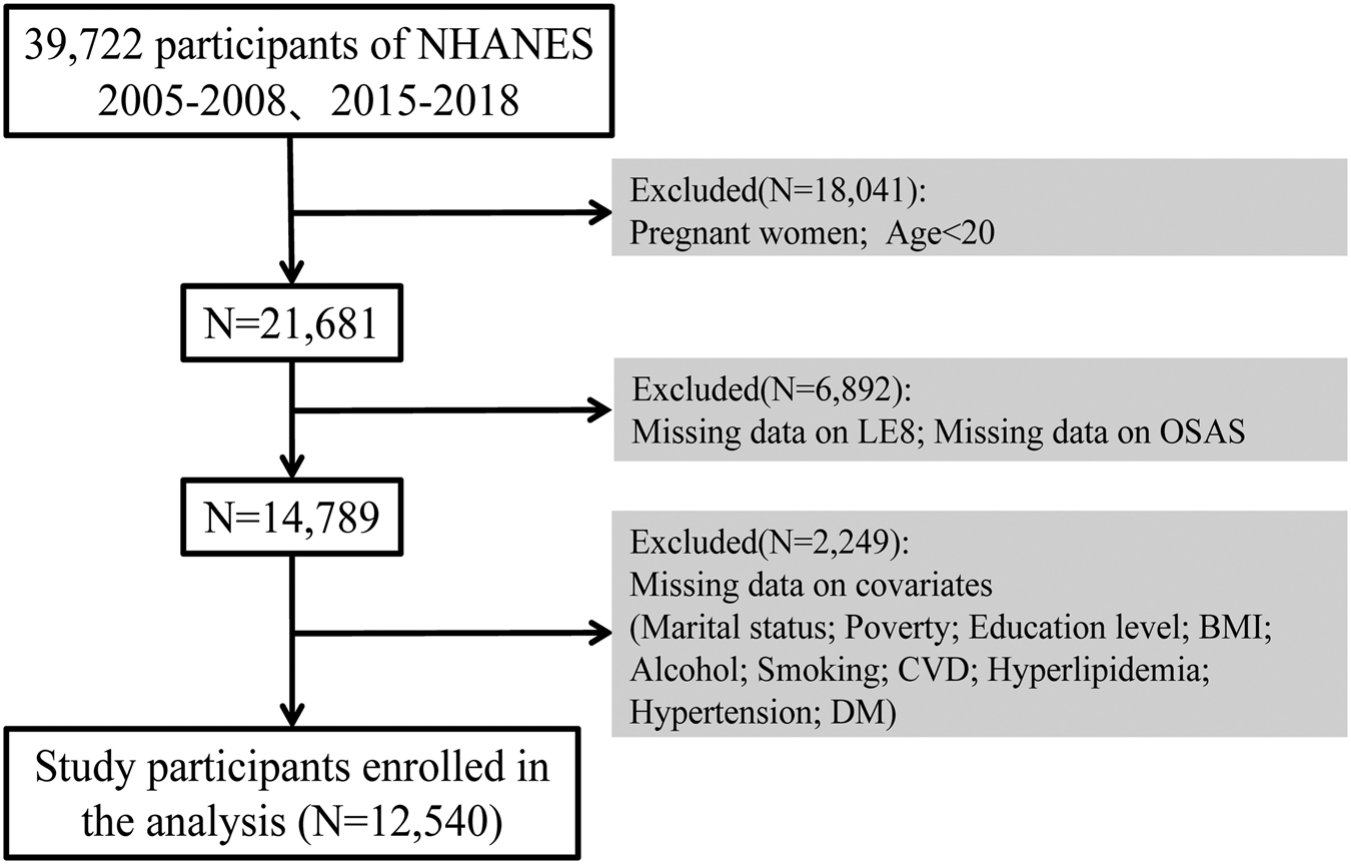
Selection of participants in the study. CVH, cardiovascular health; NHANES, national health and nutrition examination survey.

In our study, we employed data from NHANES spanning the years 2005–2008 and 2015–2018, comprising a total initial sample size of 39,722 participants. After excluding pregnant women, individuals under 20 years, and those with missing data on OSA symptoms, LE8 score-related indicators, marital status, income level, education level, BMI, alcohol consumption, smoking, and CVD, data from 12,540 participants was included in our analyses (Figure 1). We employ the List-wise Deletion method to remove entire rows that contain any missing values. This approach is advantageous due to its simplicity and ease of implementation, while also preserving the distribution of the data (Supplementary Material).

### Definition of OSA symptoms

The assessment of OSA symptoms is based on the responses to three dichotomous questions (19). The two variables measured in the study were (1) frequency of snoring, (2) frequency of snoring/breathing stops, and (3) frequency of excessive daytime sleepiness. Individuals who exhibited the following characteristics were categorized as having symptoms of OSA: snoring at least 3 times per week, snoring or interrupted breathing at least 3 times per week, and feeling excessively sleepy during the day between 16 and 30 times per month.

### Quantification of CVH

The LE8 metrics is determined by two primary criteria: health behaviors and health factors. It is specifically designed to measure an individual's overall level of CVH. The LE 8 metrics consist of the following components: dietary intake, physical activity, tobacco/nicotine exposure, sleep, BMI, HDL cholesterol, blood glucose, and blood pressure. The study employs an ordinal scoring system with a range of 0 to 100, where lower scores indicate poorer health, while higher scores indicate better health. The total LE8 score for each participant is calculated by summing the scores of all 8 components and dividing by 8. This framework includes comprehensive assessments of CVH at baseline, categorizing scores of <50 for low cardiovascular health; total scores of 50–79 for moderate cardiovascular health, and ≥80 for high cardiovascular health (20–22).

### Covariates

The covariates examined in this study encompass a range of factors, including age, gender (male and female), race and ethnicity (non-Hispanic black, non-Hispanic white, Mexican American, other Latino, and other races-including multiracial), marital status (married/living with a partner, never married, widowed/divorced/separated), personal income ratios [categorized as low income (1.3), middle income (1.3–3.49), and high income (≥3.5)], an education level (<high school, high school, college, or higher), BMI, alcohol, smoking, CVD, diabetes mellitus (DM), hypertension and hyperlipidemia (23–25).

We divided the smoking status into three categories: never smokers (less than 100 cigarettes in a lifetime), former smokers (quit after smoking more than 100 cigarettes), and current smokers. We categorized alcohol abuse status into three categories: never drinkers (less than 12 drinks in lifetime), former drinkers (less than 12 drinks in 1 year and no drinks in the last year, or no drinks in the last year but more than 12 drinks in lifetime), and currently drinking. CVD status refers to the existence or nonexistence of any of the subsequent diseases: CHD, congestive heart failure, heart attack, stroke, or angina. The American Diabetes Association criteria and self-report questionnaires define DM, and we consider a case of DM if any of the following conditions applies: (1) Doctor has informed you that you have diabetes; (2) Your glycated hemoglobin HbA1c level is equal to or greater than 6.5%; (3) The fasting blood glucose level should equal or exceed 7.0 mM/L.; (4) The random blood glucose level should equal or exceed 11.1 mM/L. (5) The two-hour OGTT blood glucose level should equal or exceed 11.1 mM/L. (6) Use of diabetes medicine or insulin.

Hypertension was assessed based on blood pressure measures taken during the physical examination, where systolic blood pressure was equal to or more than 140 mm Hg, or diastolic blood pressure was equal to or greater than 90 mm Hg. Moreover, It also needs to be determined by a physician's diagnosis of hypertension or receipt of hypertension. Definitions of hyperlipidemia include: (1) having a triglyceride level equal to or greater than 150 mg/dl.; (2) increased total cholesterol can be diagnosed if the triglyceride level is equal to or greater than 200 mg/dl, the LDL cholesterol level is equal to or greater than 130 mg/dl, or the HDL cholesterol level is below 40 mg/dl for males or 50 mg/dl for females.; and (3) The use of lipid-lowering medicines is also considered a factor in diagnosing these conditions.

### Statistical analyses

Continuous variables were presented as mean and standard deviation (SD), whereas categorical variables were displayed as frequency and weighted percentage. We used the Chi-square test to assess categorical data and the *T*-test to compare group differences in continuous variables.Our research aimed to determine the association between CVH and OSA symptoms. CVH is measured by LE 8 metrics (classified into high, medium, and low CVH groups). Participants in the low CVH group served as the reference category. We used a weighted logistic regression model to generate odds ratios (ORs) with 95% confidence intervals (CIs),taking into account into following adjustment variables: age, sex, race, marital status, poverty, education level, BMI, alcohol, smoking, CVD, DM, hyperlipidemia, and hypertension. In Crude model, we did not adjust for any confounders. In model 1, we adjusted for age, sex, and race. In model 3, we additionally adjusted for marital_status, poverty, education_level, BMI, alcohol, and smoking. In model 4, we additionally adjusted for CVD, DM, and hypertension, hyperlipidemia.

In addition, subgroup analyses were performed to investigate if the associations differed by sex (male and female) and age group (<60 and ≥60). Subgroup analyses were performed using stratified logistic regression models. The modifications and interactions of subgroups were inspected by likelihood ratio tests (26). Each stratification adjusted for the factors (race, marital_status, poverty, education_level, BMI, alcohol, smoking, CVD, DM, hypertension and hyperlipidemia). RCS was employed in weighted logistic regression models to examine the nonlinear relationships between the continuous LE8 and OSAS [Knots = 4, Reference: median (LE8) = 66.25].The selection of the number and location of knots in the RCS was guided by the Akaike information criterion (AIC) to strike a balance between optimal fit and overfitting (27).

The data were analyzed using R software version 4.2.2 provided by the R Statistical Computing Project and EmpowerStats (http://www.empowerstats.com). A two-sided test was applied, and statistical significance was defined as a *P* value < 0.05.the *P* value < 0.05 was considered statistically significant.

## Results

### Population characteristics

The baseline characteristics of the 12,540 participants in the study sample based on the weighted analyses are presented in Table 1. The sample comprised 6,360 (51.64%) females and 6,180 (48.36%) males. Among the participants, 3,785 participants (30.18%) had OSA symptoms, whereas 8,755 participants (69.82%) without OSA symptoms. Of the total participants, 8,222 (74.20%) were below 60 years old, and 4,318 (25.80%) were above 60 years old. Mexican Americans accounted for 7.40%, non-Hispanic black people accounted for 9.67%, non-Hispanic white people accounted for 71.77%, and the remaining accounted for other ethnicities, totaling 11.15%. The high CVH group consists of 2,272 participants, of whom 82.79% (1,881) did not exhibit OSA symptoms. In the moderate CVH group, there were 8,687 subjects, with 69.10% (6,003) not exhibiting OSA symptoms. The low CVH group had only 55.09% of participants without OSA symptoms. The mean LE8 score for subjects with OSA symptoms was 63.68, significantly lower than the 70.12 observed in the group without OSA symptoms (*P* < 0.001). As depicted in Table 1 participants with OSA symptoms were predominantly male, under 60 years old, married, highly educated, and former or current smokers. In addition, participants with OSA symptoms tend to have a BMI of 30 or higher, reported alcohol consumption, and did not have a history of CVD, DM, hyperlipidemia or hypertension.

**Table 1:** Characteristics by cardiovascular health level.

Variable	Total (*N* = 12,540)	No-OSAS (*N* = 8,755)	OSAS (*N* = 3,785)	*P* value
Age				0.990
<60	8,222 (74.20)	5,700 (74.19)	2,522 (74.21)	
≥60	4,318 (25.80)	3,055 (25.81)	1,263 (25.79)	
Sex				<0.0001
Female	6,360 (51.64)	4,641 (54.60)	1,719 (44.92)	
Male	6,180 (48.36)	4,114 (45.40)	2,066 (55.08)	
Race				0.600
Mexican american	2,008 (7.40)	1,432 (7.60)	576 (6.96)	
Non-hispanic black	2,539 (9.67)	1,785 (9.71)	754 (9.60)	
Non-hispanic white	5,800 (71.77)	4,027 (71.51)	1,773 (72.38)	
Others	2,193 (11.15)	1,511 (11.19)	682 (11.06)	
Marital_status				<0.0001
Divorced	1,410 (10.32)	981 (10.40)	429 (10.13)	
Living with partner	1,034 (8.18)	713 (8.13)	321 (8.32)	
Married	6,717 (57.67)	4,524 (55.75)	2,193 (62.05)	
Never married	2,039 (16.16)	1,548 (17.78)	491 (12.46)	
Separated	389 (2.25)	266 (2.09)	123 (2.62)	
Widowed	951 (5.41)	723 (5.85)	228 (4.41)	
Education_level				0.010
High school	2,974 (24.16)	2,029 (23.06)	945 (26.65)	
Less than High school	2,778 (13.30)	1,969 (13.27)	809 (13.38)	
More than High school	6,788 (62.54)	4,757 (63.67)	2,031 (59.97)	
Poverty				0.840
≤1	2,251 (11.24)	1,579 (11.28)	672 (11.13)	
>1	10,289 (88.76)	7,176 (88.72)	3,113 (88.87)	
BMI				<0.0001
<25	3,470 (29.35)	2,835 (34.79)	635 (16.97)	
≥25 to <30	4,216 (32.73)	3,014 (33.39)	1,202 (31.23)	
≥30	4,854 (37.92)	2,906 (31.82)	1,948 (51.80)	
Alcohol				<0.0001
Former	1,980 (12.59)	1,361 (11.89)	619 (14.18)	
Heavy	2,408 (20.32)	1,621 (19.45)	787 (22.29)	
Mild	4,426 (38.69)	3,046 (38.79)	1,380 (38.45)	
Moderate	2,051 (18.32)	1,423 (18.38)	628 (18.19)	
Never	1,675 (10.09)	1,304 (11.49)	371 (6.89)	
Smoking				<0.0001
Former	3,211 (25.97)	2,147 (24.96)	1,064 (28.27)	
Never	6,825 (54.67)	4,990 (57.27)	1,835 (48.76)	
Now	2,504 (19.35)	1,618 (17.76)	886 (22.97)	
CVD				<0.001
No	11,141 (91.78)	7,864 (92.56)	3,277 (90.01)	
Yes	1,399 (8.22)	891 (7.44)	508 (9.99)	
Hyperlipidemia				<0.0001
No	3,551 (29.57)	2,665 (31.97)	886 (24.09)	
Yes	8,989 (70.43)	6,090 (68.03)	2,899 (75.91)	
Hypertension				<0.0001
No	7,320 (63.65)	5,376 (67.20)	1,944 (55.57)	
Yes	5,220 (36.35)	3,379 (32.80)	1,841 (44.43)	
DM				<0.0001
No	10,455 (87.58)	7,494 (89.39)	2,961 (83.48)	
Yes	2,085 (12.42)	1,261 (10.61)	824 (16.52)	
LE8_Group				<0.0001
High_CVH	2,272 (21.87)	1,881 (26.03)	391 (12.41)	
Low_CVH	1,581 (9.95)	871 (7.51)	710 (15.48)	
Moderate_CVH	8,687 (68.18)	6,003 (66.46)	2,684 (72.11)	
LE8	68.15 (0.36)	70.12 (0.36)	63.68 (0.42)	<0.0001

Mean (sd) for continuous; *n* (%) for categorical (Percent values were weighted to account for the complex survey design.).

*T*-test for continuous; chi-squared test for categorical.

### Associations of Le8 with OSA

The results of the sample-weighted multivariate logistics regression analyses are presented in Table 2. Across all models, there was a significant inverse correlation between symptoms of OSA and LE8 metrics. The 95% CIs for the crude model, model 1, model 2, and model 3 were 0.968 (0.964,0.971), 0.967 (0.963, 0.971), 0.979 (0.974, 0.985), and 0.983 (0.978, 0.988), respectively, all with *P* < 0.0001. After adjustment for multiple covariates, the 95% CI in model 3 was 0.750 (0.630, 0.893) for the moderate CVH group and 0.573 (0.454,0.723) for the high CVH group. The trend *P*-value for all models was less than 0.0001, indicating a significant decrease in the risk of developing OSA symptoms with increasing LE8 scores.

**Table 2:** Crude and adjusted association between life's essential 8 score and obstructive sleep apnea symptoms.

Character	Crude model		Model 1		Model 2		Model 3	
	95% CI	*P*	95% CI	*P*	95% CI	*P*	95% CI	*P*
LE8	0.968 (0.964,0.971)	<0.0001	0.967 (0.963,0.971)	<0.0001	0.979 (0.974,0.985)	<0.0001	0.983 (0.978,0.988)	<0.0001
LE8_Group								
Low_CVH	ref		ref		ref		ref	
Moderate_CVH	0.527 (0.453,0.612)	<0.0001	0.508 (0.435,0.593)	<0.0001	0.671 (0.564,0.798)	<0.0001	0.750 (0.630,0.893)	0.002
High_CVH	0.231 (0.193,0.278)	<0.0001	0.227 (0.188,0.273)	<0.0001	0.474 (0.372,0.605)	<0.0001	0.573 (0.454,0.723)	<0.0001
*P* for trend		<0.0001		<0.0001		<0.0001		<0.0001

Model 1: age, sex, race.

Model 2: age, sex, race, marital_status, poverty, education_level, BMI, alcohol, smoking.

Model 3: age, sex, race, marital_status, poverty, education_level, BMI, alcohol, smoking, CVD, DM, hypertension, hyperlipidemia.

### Subgroup analyses

Subgroup analyses by age and gender in Table 3 specifically examine the relationship between LE8 scores and OSA. The results demonstrate a significant interaction between LE8 scores and OSA symptoms with age (*P* < 0.0001). The high cardiovascular health (CVH) group exhibited a lower risk of concurrent OSA symptoms (OR: 0.470; 95% CI: 0.345, 0.641) (Table 4). In the subgroup of male participants, lower LE8 scores were associated with OSA symptoms (OR: 0.982; 95% CI: 0.974, 0.989). In the subgroup of participants aged less than 60 years, lower LE8 scores were significantly associated with OSA symptoms (OR: 0.980; 95% CI: 0.973, 0.986) (Table 3).

**Table 3:** Associations of life's essential 8 score for obstructive sleep apnea symptoms grouped by age, sex.

Character	OR (95% CI)	*P*	*P* for interaction
Age			<0.0001
<60	0.980 (0.973,0.986)	<0.0001	
≥60	0.994 (0.985,1.003)	0.198	
Sex			0.348
Female	0.986 (0.979,0.993)	<0.001	
Male	0.982 (0.974,0.989)	<0.0001	

Adjust for race, marital_status, poverty, education_level, BMI, alcohol, smoking, CVD, DM, hypertension and hyperlipidemia.

**Table 4:** The high cardiovascular health group showed a lower risk of developing OSA symptoms.

Character	Low_CVH	Moderate_CVH	*P*	High_CVH	*P*	*P* for trend	*P* for interaction
Age							<0.001
<60	ref	0.623 (0.503,0.772)	<0.0001	0.470 (0.345,0.641)	<0.0001	<0.0001	
≥60	ref	1.027 (0.781,1.352)	0.844	0.952 (0.584,1.551)	0.839	0.894	
Sex							0.461
Female	ref	0.861 (0.659,1.126)	0.266	0.783 (0.540,1.135)	0.19	0.194	
Male	ref	0.663 (0.517,0.850)	0.002	0.425 (0.304,0.595)	<0.0001	<0.0001	

Adjust for race, marital_status, poverty, education_level, BMI, alcohol, smoking, CVD, DM, hypertension and hyperlipidemia.

In summary, for men under 60 years of age, it was shown that those with high CVH grades had a notably reduced risk of experience concomitant OSA symptoms in comparison to those with low CVH grades. There was a notable correlation between the risk ratios and increasing CVH grade, suggesting that CVH grade is a significant risk factor in OSA. A substantial correlation was seen between age and CVH grade, suggesting that age may influence the relationship between CVH grade and OSA.

### Dose-response analysis of Le8 score with OSA

**Figure 2:**
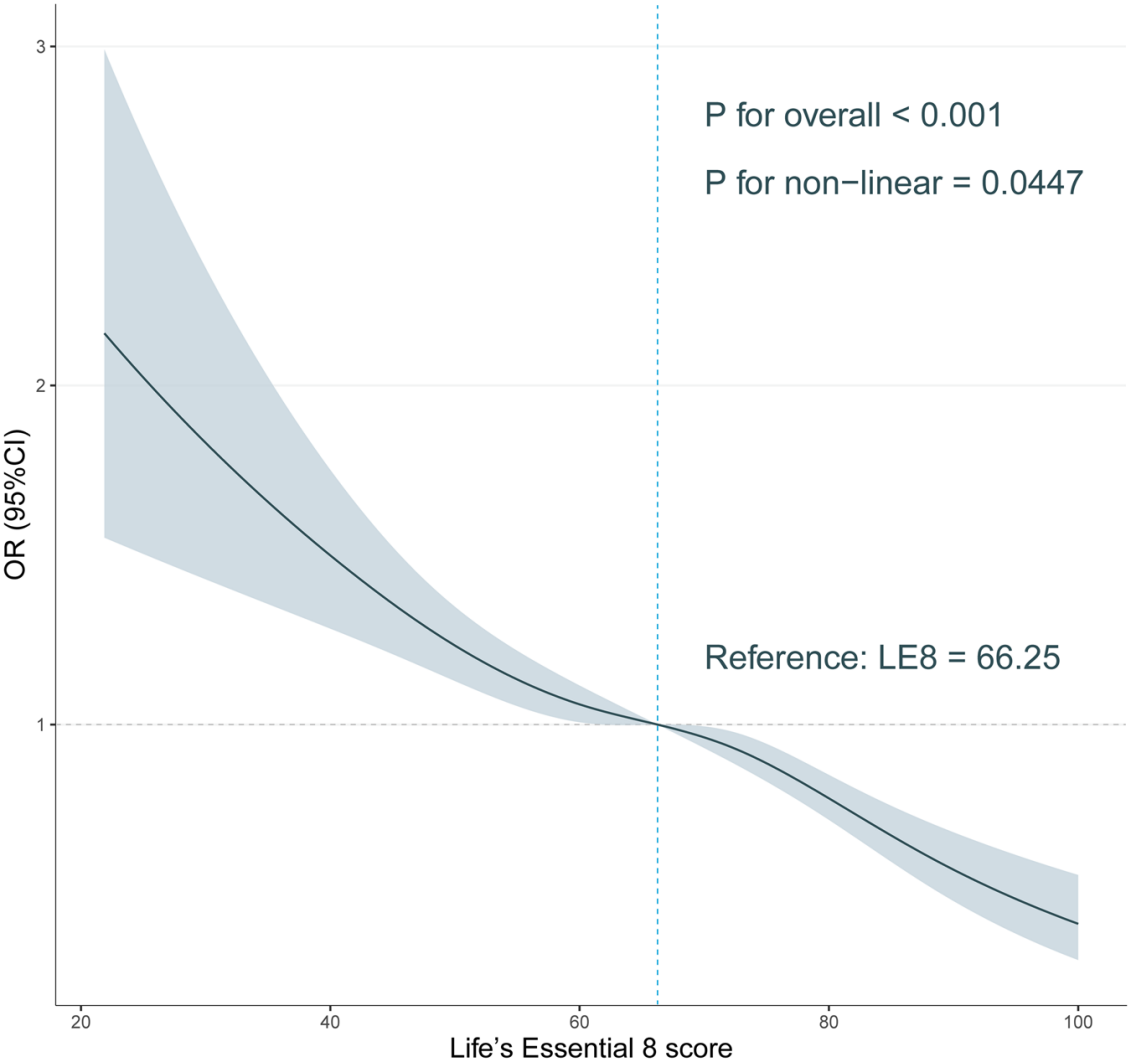
Association of Life’s Essential 8 score with obstructive sleep apnea symptoms in a restricted cubic spline model among all participants.

Multivariate corrected RCS analyses indicated a significant inversely nonlinear relationship between LE8 score and OSA (*P* for nonlinear = 0.0447; Figure 2). Our finding demonstrates a substantial decrease in OSA symptom prevalence with increased LE 8 scores.

## Discussion

Our study is one of the few to use the NHANES database to conduct a cross-sectional analysis to explore and understand the intrinsic relationship between CVH and OSA. We have observed that CVH subgroups may have a strong association with OSA symptoms, even after adjusting for confounders. The analyses conducted on 12,540 participants revealed that LE8 scores have a negative correlation with the proportion of OSA symptoms. This association was consistent across various demographic characteristics, including gender, race and ethnicity, income, and marital status. However, there was a significant interaction with age. Our findings highlight the importance of maintaining a high CVH to prevent OSA, echoing findings from Zhang et al. (28).

OSA is a common systemic disease that is closely related to diseases such as CHD and DM, as well as potentially causing sudden death and contributing to road traffic accidents, serving as a serious social problem along with a serious healthcare burden for the public (13, 29, 30). Individuals with OSA experience repeated upper airway obstruction and apnea during sleep, which may lead to a variety of cardiovascular problems, such as decreased oxygen saturation during sleep, a known risk factor for hypertension (31). Häusler et al. identified an association between sleep-related factors such as OSA and an elevated risk of CVD events, though the exact nature of this relationship remains incompletely understood (32).Recent research reveals a complex interplay among intermittent hypoxia, oxidative stress, inflammation, and autonomic dysregulation, which collectively contribute to increased cardiovascular risk in patients with OSA (33). Therefore, given the incomplete investigation of the association and the existing gap in evidence in the US, our cross-sectional study provides essential findings to contribute to the existing knowledge base.

From the analysis of the 12,540 participants included in the study, we found that the population with concomitant OSA symptoms was characterized by being male, having a BMI ≥30, having an alcohol consumption history, and being a former or current smoker (34). The prevalence of participants with concomitant OSA symptoms was notably higher among those diagnosed with CVD, hyperlipidemia, hypertension, or DM (*P* < 0.0001). This underscores the interconnectedness and shared risk factors among these conditions, such as unhealthy lifestyles, poor dietary habits, obesity, and smoking (35). We applied the LE8 scoring system to facilitate the quantitative assessment of participants' CVH status. The average LE 8 score was 63.68 in the group with OSA symptoms, which was significantly lower than the 70.12 in group without OSA symptoms (*P* < 0.0001). The proportion of participants with concomitant OSA symptoms was considerably higher in low CVH compared to the moderate CVH and high CVH groups. We analyzed the included participants in subgroups by age and gender and found a significant interaction between OSA symptoms with age and LE8 score (*P* < 0.0001). In subjects under 60 years of age, a reduced risk of concomitant OSA symptoms was observed in the high CVH group (OR: 0.470; 95% CI: 0.345, 0.641). The OR showed a significant increase with a higher CVH grade, indicating a significant interaction between age and CVH grading. For those under 60 years of age, maintaining a high LE 8 score significantly reduces the probability of developing OSA symptoms. However, this preventive effect appears less pronounced in individuals aged 60 and above. It is widely acknowledged that the risk of OSA may escalate with age, therefore, further research into preventive approaches aimed at mitigating OSA risk among older people becomes vital (36).

Our study is based on the NHANES database, selecting eligible samples for weighted analysis to explore the association between CVH and OSA. This finding can be extrapolated to the U.S. adult population-offering health policy insights and serving as a foundation for further research in public health interventions. Considering the interrelationship between CVH and OSA, enhancing public awareness and promoting CVH-related diagnosis and treatment may also be an effective means to improve OSA, which serves as an intervention that aims to address potential CVH issues and improve oxygen saturation (32, 37). At the same time, maintaining healthy lifestyle habits also serves as an effective method to improve OSA and CVH.

## Limitation

This study also has some limitations. First, the identification of OSA symptoms relied primarily on the typical clinical presentations on the questionnaire, which may not capture all aspects of the condition. Some of the symptoms that are crucial for identifying OSA were not available, such as lifestyle behaviors, which may have resulted in some OSA participants not being identified (38). Moreover, using questionnaire-based assessments alone may overlook certain cases of OSA. Second, our study is limited to factors such as snoring, intermittent apnea, and drowsiness. This limitation could potentially result in some individuals with OSA not being identified (39). Third, the participants from the database lacked relevant information about professional laboratory sleep testing, potentially leaving out individuals with asymptomatic OSA from the OSA group. However, these missing individuals are likely to be undifferentiated and have no effect on the final analysis results (40). Fourth, our study is constrained by its retrospective and cross-sectional design, which introduces information bias stemming from missing data and complicates the establishment of a causal relationship between LE8 scores and OSA. Additionally, we did not employ Directed Acyclic Graphs (DAGs) to identify and elucidate potential confounding variables. Future research will concentrate on this aspect.

## Conclusion

The findings of the research conducted on a large and diverse group of 12,540 individuals in the United States, using data from the NHANES, indicate a strong negative association between LE8 scores and OSA symptoms. Participants with higher LE8 scores were less likely to have concomitant OSA symptoms. These findings underscore the importance of adopting a healthy lifestyle and maintaining a high LE8 score to reduce the incidence of OSA symptoms and their potential role in preventing OSA-related complications.Future research should concentrate on investigating the causal relationships and clarifying the precise mechanisms underlying the relationship between CVH and OSA.

## Data Availability

The original contributions presented in the study are included in the article/Supplementary Material, further inquiries can be directed to the corresponding author.
